# Comparison of Perioperative Risk Assessment Tools for Major Postoperative Complications After Radical Cystectomy

**DOI:** 10.3390/diagnostics16213439

**Published:** 2026-10-22

**Authors:** Ahmet Halil Sevinç, Burcu Hancı Sevinç, Erdinç Dinçer, Osman Murat İpek, Alparslan Çetin, Cengiz Çanakcı, Orkunt Özkaptan, Antonios Koudonas

**Affiliations:** 1 Department of Urology, Kartal Dr. Lütfi Kırdar City Hospital, 34865 Istanbul, Türkiye; burcuhanci91@hotmail.com (B.H.S.);; 2 First Department of Urology, School of Medicine, Aristotle University of Thessaloniki, 54124 Thessaloniki, Greece

**Keywords:** bladder cancer, radical cystectomy, perioperative risk, postoperative morbidity, risk stratification, frailty

## Abstract

**Background/Objectives**: To compare the discriminatory performance of commonly used perioperative risk assessment tools, including the American Society of Anesthesiologists (ASA) Physical Status Classification, Estimation of Physiologic Ability and Surgical Stress (E-PASS), Surgical Outcome Risk Tool (SORT), and modified 5-item Frailty Index (mFI-5), for major postoperative complications after radical cystectomy. **Methods**: This retrospective observational study included 193 patients who underwent open radical cystectomy for bladder cancer between January 2017 and January 2025. Major postoperative complications were defined as Clavien–Dindo grade III or higher. The discriminatory performance of ASA, E-PASS Comprehensive Risk Score (CRS), SORT, and mFI-5 was assessed using receiver operating characteristic analysis. Pairwise comparisons of the areas under the receiver operating characteristic curves (AUCs) were performed using the DeLong test. Logistic regression models were used to evaluate associations with major complications, and exploratory analyses examined secondary postoperative outcomes. **Results**: Major postoperative complications occurred in 58 (30.1%) patients. The numerical AUC was highest for E-PASS CRS (0.618, 95% CI 0.536–0.701), followed by SORT (AUC 0.610, 95% CI 0.525–0.694), ASA (AUC 0.595, 95% CI 0.509–0.680) and mFI-5 (AUC 0.578, 95% CI 0.489–0.668). However, pairwise DeLong tests showed no statistically significant difference in discrimination between E-PASS CRS and SORT, or any of the other scores tested. In univariable analysis, high E-PASS CRS, high SORT score and mFI-5 ≥ 2 were significantly associated with major complications, while ASA classification was not. When all scores were entered into the multivariable model at the same time, none of the scores remained statistically significant. **Conclusions**: All of the risk assessment tools evaluated in this study demonstrated poor discriminatory ability for major postoperative complications following radical cystectomy. Although E-PASS CRS had the highest numeric AUC, its discrimination was not statistically better than the other scores analyzed. Existing risk tools may help with perioperative risk stratification but should not be used as stand-alone prediction models for major postoperative morbidity after radical cystectomy.

## 1. Introduction

Bladder cancer (BC) is the seventh most common cancer worldwide and the second most common urological malignancy [1]. In 2020, approximately 573,000 new cases and 213,000 BC-related deaths were reported globally, with substantial variation in incidence and mortality according to sex and geographic region [2]. The global burden of BC is expected to increase further with population growth and aging [1,2].

Radical cystectomy (RC) remains a standard treatment for muscle-invasive bladder cancer (MIBC) and selected patients with high-risk non-muscle-invasive disease [3]. Despite advances in surgical techniques and perioperative care, RC is associated with considerable postoperative morbidity. Approximately 45% and 15% of patients experience minor (Clavien–Dindo I–II) and major (Clavien–Dindo III–V) complications, respectively, with gastrointestinal and infectious complications being among the most frequent events [4]. Although postoperative outcomes have improved over time [5], the substantial morbidity of RC remains an important consideration in patient selection, particularly in patients with limited physiological reserve. Importantly, chronological age alone may not adequately reflect surgical fitness, as favorable postoperative outcomes have also been reported in carefully selected octogenarians undergoing RC [6].

Accurate perioperative risk stratification may therefore improve patient selection and perioperative management. Several scoring systems have been developed to estimate postoperative morbidity and mortality, including the American Society of Anesthesiologists (ASA) Physical Status Classification System [7], Estimation of Physiologic Ability and Surgical Stress (E-PASS) [8], Surgical Outcome Risk Tool (SORT) [9], and modified 5-item Frailty Index (mFI-5) [10]. These tools differ in the extent to which they incorporate physiological reserve, frailty, and surgical stress, and their comparative performance specifically in patients undergoing RC remains uncertain. Importantly, these instruments are not conceptually equivalent, with ASA, SORT and mFI-5 relying mostly on pre-operative patient characteristics, while the E-PASS Comprehensive Risk Score (CRS) combines pre-operative physiological factors with intra-operative markers of surgical stress. Therefore, the scores obtained with these tools should be regarded as perioperative risk estimators and not as directly comparable preoperative prediction models. Therefore, the aim of the present study was to assess the comparative discriminatory capacity of ASA, E-PASS CRS, SORT and mFI-5 for major complications after RC and to explore whether any of these commonly used risk assessment tools provide clinically relevant discrimination in this patient population.

## 2. Materials and Methods

### 2.1. Study Design and Patient Selection

This retrospective observational study included adult patients who underwent radical cystectomy for bladder cancer at Kartal Dr. Lütfi Kırdar City Hospital between 1 January 2017 and 1 January 2025. A total of 222 patients who underwent radical cystectomy were initially evaluated for eligibility during the study period. Patients with missing perioperative data for the risk score calculation (*n* = 20), cystectomy for benign conditions (*n* = 3), inflammatory bowel disease (*n* = 2), prior abdominal or pelvic irradiation (*n* = 2), or neoadjuvant chemotherapy (*n* = 2) were excluded from the study, resulting in 29 patients being excluded. Complete-case analysis was used to deal with missing data to ensure accuracy. Thus, the final analytical group comprised 193 patients who underwent open radical cystectomy with ileal conduit diversion.

Data were obtained from electronic medical records, anesthesiology records, operative records, and postoperative follow-up files. Patients aged 18 years or older with available perioperative and follow-up data were eligible. Patients undergoing cystectomy for non-malignant indications, emergency surgery, or those with insufficient data required for risk-score calculation or outcome assessment were excluded.

The study was conducted in accordance with the principles of the Declaration of Helsinki and was approved by the Scientific Research Ethics Committee of Kartal Dr. Lütfi Kırdar City Hospital (approval no: 2026/010.99/30/30, 22 July 2026) for the retrospective analysis of procedures performed between January 2017 and January 2025. The study was retrospective and thus an obligation for informed consent was waived according to local regulations.

### 2.2. Data Collection

The following preoperative variables were recorded: age, sex, body mass index, American Society of Anesthesiologists (ASA) physical status classification, Eastern Cooperative Oncology Group (ECOG) performance status, and comorbidities including hypertension, diabetes mellitus, coronary artery disease, chronic obstructive pulmonary disease, congestive heart failure, chronic kidney disease, cerebrovascular disease, and endocrine disorders.

All procedures were performed using an open surgical approach. Standard pelvic lymph node dissection was routinely performed in all patients, and an ileal conduit was used as the urinary diversion in every case. Intraoperative variables included operative time, estimated blood loss normalized to body weight, and perioperative blood transfusion. Estimated blood loss was obtained from operative and anesthesiology records.

Postoperative variables included Clavien–Dindo complication grade, intensive care unit (ICU) requirement, ICU length of stay, total hospital stay, readmission, and mortality at 30 days, 90 days, and 1 year after surgery. Postoperative complications were retrospectively identified from electronic medical records.

### 2.3. Outcome Measures

The main outcome measure was major postoperative complications defined as Clavien–Dindo grade III or higher within 90 days after the surgery. Postoperative complications were retrospectively identified by reviewing electronic medical records, operative reports, and follow-up notes. Complications after discharge were recorded systematically by scheduling outpatient visits to the urology clinic, emergency room visits and hospital readmissions within 90 days. In addition, linkage to the national electronic health record system allowed confirmation of admissions and surgical/interventional procedures performed at outside facilities during the follow-up period.

Secondary outcomes included ICU requirement, ICU length of stay, total hospital stay, readmission, and all-cause mortality at 30 days, 90 days, and 1 year after surgery.

Survival status was verified using hospital electronic medical records, the national electronic health record system, the national death registry, and telephone contact when necessary.

### 2.4. Risk Score Calculation

For each patient, the ASA Physical Status Classification, E-PASS score, Surgical Outcome Risk Tool (SORT), and modified 5-item Frailty Index (mFI-5) were calculated.

ASA Physical Status: Obtained directly from verified pre-induction records completed by board-certified anesthesiologists without retrospective reclassification.

The E-PASS includes the Preoperative Risk Score (PRS), Surgical Stress Score (SSS), and Comprehensive Risk Score (CRS) calculated according to the original validated formulas of Haga et al. [8]. The PRS comprises six preoperative physiologic variables: age, major cardiac disease (NYHA functional class III–IV or history of myocardial infarction < 6 months), major pulmonary disease (COPD severe, FEV1 < 50% or dyspnea at rest), diabetes mellitus, performance status (ECOG) and ASA class. The SSS was based on intra-operative variables including blood loss normalized to body weight (g/kg, 1 mL = 1 g), total operative time (hours) and incision category (0, minor or laparoscopic/thoracoscopic approach; 1, laparotomy or thoracotomy alone; 2, both laparotomy and thoracotomy). As all procedures involved laparotomy alone, the incision category was scored as 1. Subsequently, the comprehensive perioperative risk (CRS) was defined based on the PRS and SSS.

The predicted risk of 30-day postoperative mortality was calculated using the SORT model developed and validated by Protopapa et al. [9], which incorporates patient age, ASA physical status, surgical urgency, surgical severity, high-risk surgical specialty, and cancer status. In this study, all procedures were classified as expedited, reflecting the perceived need for oncologic surgery within days to weeks. Surgical severity was classified as major/complex because all patients underwent radical cystectomy with urinary diversion. The high-risk surgical specialty variable was coded as present on the basis of extensive pelvic surgery and gastrointestinal reconstruction using an ileal conduit. Cancer status was coded as present in all patients.

The mFI-5 was calculated by assigning one point for each of five criteria (total possible score, 0–5): hypertension requiring medication; diabetes mellitus treated with insulin or oral hypoglycemic agents; a history of chronic bronchitis, emphysema, or chronic obstructive pulmonary disease; congestive heart failure within 30 days before surgery; and functional dependence [10]. ECOG performance status ≥ 2 was used as a surrogate for functional dependence, consistent with a previously reported approach [11]. Patients were classified as non-frail (mFI-5 < 2) or frail (mFI-5 ≥ 2) for comparative analyses.

Statistical analyses were performed using IBM SPSS Statistics for Windows, version 23.0 (IBM Corp., Armonk, NY, USA). The distribution of continuous variables was assessed using the Shapiro–Wilk test. Normally distributed variables were expressed as mean ± standard deviation, whereas non-normally distributed variables were expressed as median and interquartile range (IQR). Categorical variables were presented as frequencies and percentages.

Patients were compared according to the presence or absence of major postoperative complications. Continuous variables were compared using Student’s *t* test or the Mann–Whitney U test, as appropriate, and categorical variables using the chi-square test or Fisher’s exact test.

The discriminatory performance of ASA, E-PASS CRS, SORT, and mFI-5 for major postoperative complications was evaluated using receiver operating characteristic (ROC) curve analysis, and the area under the curve (AUC) with 95% confidence intervals was calculated for each score. The areas under the receiver operating characteristic curves (AUC) were assessed in pairs utilizing a nonparametric test for paired data (DeLong test) to assess whether any statistically significant differences existed in the discriminative efficacy of the four risk ratings. AUC values were evaluated in comparison to a null value of 0.50; the statistical significance of an individual AUC exceeding 0.50 was not construed as proof that one score was better than another.

Univariable logistic regression analyses were performed for each risk score. Multivariable logistic regression models were subsequently constructed by adjusting for operative time and by including all evaluated risk scores in the same model to assess their independent associations and potential collinearity. Variance Inflation Factor (VIF) analysis was used to test for multicollinearity among the intersecting risk indices. Logistic regression results for the primary outcome are reported as odds ratios (ORs) with 95% confidence intervals (CIs) and corresponding *p*-values. Model calibration was assessed using the Hosmer–Lemeshow goodness-of-fit test. A *p*-value > 0.05 was interpreted as no statistically significant evidence of lack of fit.

Exploratory secondary analyses examined the associations of the risk scores with ICU requirement, 30-day readmission, and mortality at 30 days, 90 days, and 1 year using univariable logistic regression. Associations with ICU and total hospital length of stay were evaluated using Spearman rank correlation. For secondary binary outcomes, ASA was analyzed as class III versus classes I–II, E-PASS CRS per 0.1-unit increase, SORT per 1-percentage-point increase in predicted 30-day mortality risk, and mFI-5 as ≥2 versus <2. Results are presented as odds ratios (ORs) with 95% confidence intervals. Given the relatively small number of events for several of the secondary outcomes, these analyses were deemed to be hypothesis-generating and exploratory rather than confirmatory. Multivariable models were not constructed for secondary outcomes because of the limited number of events in some outcome categories. *p*-value < 0.05 was considered statistically significant.

Patients initially assessed for eligibility

       (*n* = 222)

        │

        ▼

    Exclusions (*n* = 29):

    ├── Missing data for risk score calculation (*n* = 20)

    ├── Cystectomy for benign conditions (*n* = 3)

    ├── Inflammatory bowel disease (*n* = 2)

    ├──Previous abdominal and pelvic irradiation (*n* = 2)

    └── Neoadjuvant chemotherapy (*n* = 2)

        │

        ▼

   Final study cohort analyzed using

    complete-case analysis

       (*n* = 193)

## 3. Results

A total of 193 patients were included in the final analysis. Major postoperative complications (Clavien–Dindo grade ≥ III) occurred in 58 patients (30.1%), whereas 135 patients (69.9%) had no major complications. The demographic, clinical, and perioperative characteristics of the study population according to the presence of major postoperative complications are presented in Table 1. Among the 58 patients with major complications, the most frequently recorded categories were infectious complications (22/58, 37.9%), gastrointestinal complications (20/58, 34.5%), wound-related complications, including intervention for surgical-site infections or management of wound dehiscence (20/58, 34.5%), and renal complications, including nephrostomy placement or renal failure requiring hemodialysis (19/58, 32.8%). Because some patients experienced more than one complication, the categories were not mutually exclusive.

Patients who developed major complications had a higher prevalence of ASA class III–IV and mFI-5 ≥ 2 and higher E-PASS Comprehensive Risk Score (CRS) and SORT-predicted 30-day mortality risk. Age, sex, body mass index, ECOG performance status, and operative time did not differ significantly between the groups.

The logistic regression analyses are presented in Table 2. In the univariable models, higher E-PASS CRS, higher SORT score, and mFI-5 ≥ 2 were significantly associated with major postoperative complications, whereas ASA classification was not. In the comprehensively adjusted multivariable models (Table 2, Columns 9 and 10), the concurrent inclusion of the overlapping risk scores assessed their independent predictive significance. The reduction in individual statistical significance indicates the substantial prognostic overlap present among different composite scoring systems within our group. The Hosmer–Lemeshow test detected no statistically significant evidence of lack of fit for the multivariable models (all *p* > 0.05). VIFs were 1.73 for E-PASS CRS, 2.31 for SORT, and 1.44 for mFI-5. The higher VIFs for the ASA class II and III indicators (7.42 and 8.23, respectively) may partly reflect the limited number of patients in the ASA I reference category and overlap between ASA classification and the other risk scores.

**Figure 1:**
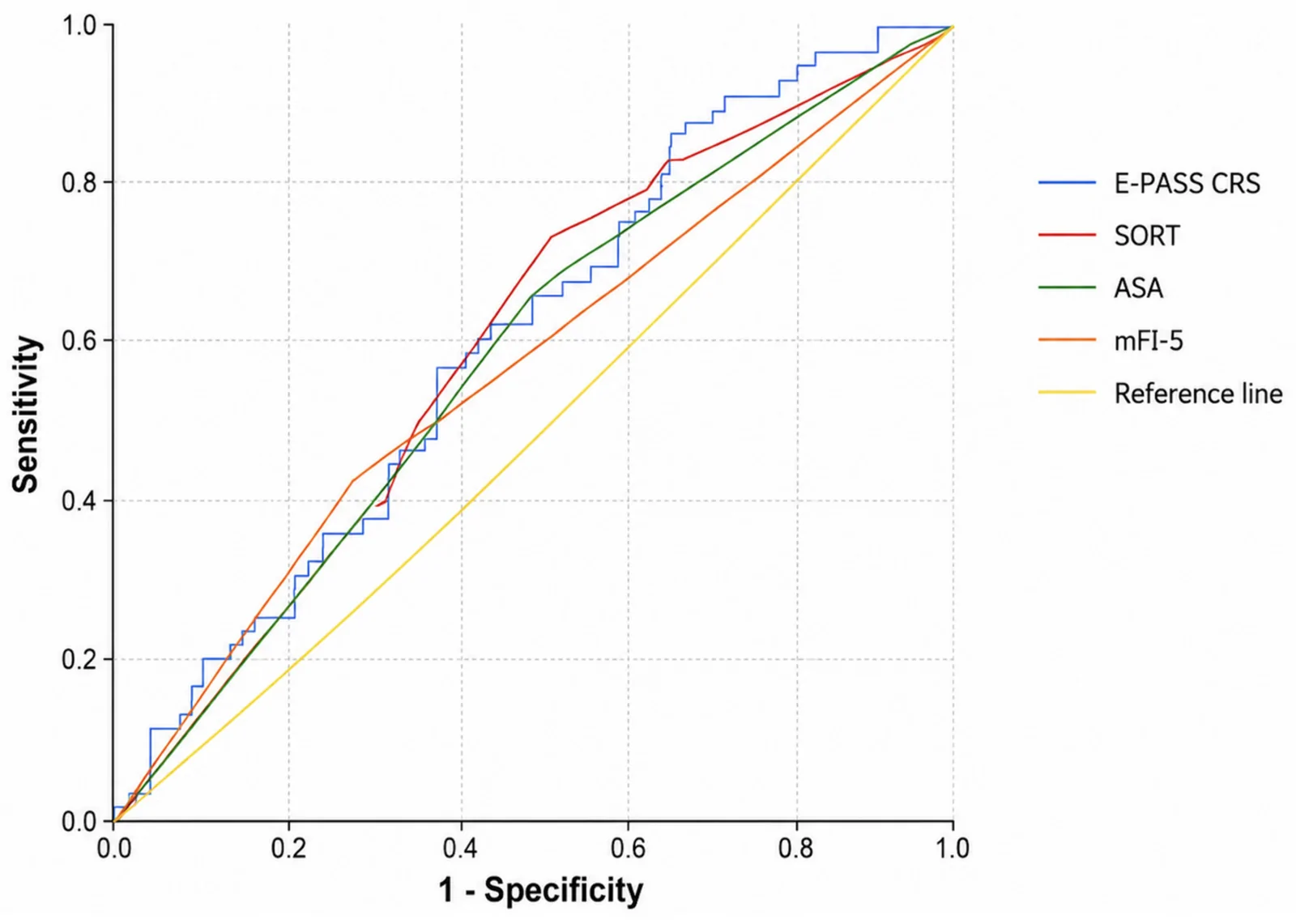
Receiver operating characteristic (ROC) curves of the evaluated risk scores in prediction of relevant postoperative complications (Clavien–Dindo grade ≥ III). The areas under the curves (AUC) are as follows: E-PASS CRS, 0.618 (95% CI, 0.536–0.701); SORT, 0.610 (95% CI, 0.525–0.694); ASA classification, 0.595 (95% CI, 0.509–0.680); and mFI-5 ≥ 2, 0.578 (95% CI, 0.489–0.668).

The discriminative ability of the four risk scores is shown in Table 3 and Figure 1. E-PASS CRS demonstrated the highest numerical AUC (0.618, 95% CI 0.536–0.701; *p* = 0.009), followed by SORT (AUC 0.610, 95% CI 0.525–0.694; *p* = 0.016), ASA (AUC 0.595, 95% CI 0.509–0.680; *p* = 0.037), and mFI-5 (AUC 0.578, 95% CI 0.489–0.668; *p* = 0.084).

Regarding secondary outcomes, 167 (86.5%) were postoperatively admitted to ICU, the median ICU length of stay was 1 day (range, 1–11), and the median total hospital length of stay was 8 days (range, 6–33). Thirty-three patients (17.1%) were readmitted within 30 days. All-cause mortality was 6/193 (3.1%) at 30 days, 8/193 (4.1%) at 90 days, and 40/193 (20.7%) at 1 year. Higher E-PASS CRS was significantly associated with 30-day readmission (OR 1.17, 95% CI 1.01–1.34; *p* = 0.031), whereas higher SORT-predicted 30-day mortality risk was significantly associated with 90-day mortality (OR 1.15, 95% CI 1.02–1.28; *p* = 0.017). ASA ≥ 3, E-PASS CRS, and mFI-5 ≥ 2 showed weak positive correlations with ICU length of stay (ρ = 0.147, *p* = 0.042; ρ = 0.154, *p* = 0.033; and ρ = 0.155, *p* = 0.032, respectively). mFI-5 ≥ 2 was also weakly and inversely correlated with total hospital length of stay (ρ = −0.151, *p* = 0.036). E-PASS CRS showed a borderline association with 1-year mortality (OR 1.14, 95% CI 1.00–1.30; *p* = 0.050) (Table 4). Considering the limited quantity of events and the investigative nature of these analyses, individual associations should be interpreted cautiously and do not indicate reliable predictive correlations between the risk scores and subsequent postoperative outcomes. No singular risk score was uniformly linked to all evaluated secondary surgical outcomes. The paired comparison utilizing the DeLong test did not indicate any notable difference between E-PASS CRS and SORT (*p* = 0.841) or any other score pairs (all *p* > 0.05) (Table 5). Consequently, the superior AUC of E-PASS CRS should not be construed as a sign of enhanced discrimination efficacy.

## 4. Discussion

In the present study, we compared the discriminative ability of ASA, E-PASS, SORT, and mFI-5 for major postoperative complications after radical cystectomy. None of the evaluated risk scores demonstrated adequate discrimination in this cohort. The numerical AUC was highest for E-PASS CRS (0.618), followed by SORT (0.610), ASA (0.595), and mFI-5 (0.578); nevertheless, pairwise DeLong testing did not reveal any statistically significant differences in discrimination among the evaluated scores. In univariable analyses, E-PASS CRS, SORT, and mFI-5 ≥ 2 were significantly associated with major postoperative complications, whereas ASA classification was not. These associations were materially unchanged after adjustment for operative time. When all risk scores were entered simultaneously into the multivariable models, none remained statistically significant, suggesting substantial overlap in the clinical information captured by these scoring systems. Collectively, these results do not endorse the dominance of any individual assessed score and instead indicate that frequently utilized generic risk assessment instruments possess restricted capacity to differentiate patients susceptible to significant postoperative complications following radical cystectomy.

Radical cystectomy remains associated with substantial perioperative morbidity despite improvements in surgical technique and perioperative care [3,5,12]. Accurate risk stratification is therefore important for patient counseling, perioperative planning, and identification of patients who may benefit from intensified perioperative management. Nevertheless, most commonly used risk scores were originally developed in heterogeneous surgical populations rather than specifically in patients undergoing radical cystectomy. This may partly explain their limited discriminatory ability in this setting. Adamczyk et al. similarly compared several risk assessment systems in patients undergoing radical cystectomy and found that none provided sufficiently accurate prediction of major postoperative complications [13].

Consistent with our findings, Covotta et al. also reported the numerically highest AUC for E-PASS among four scores in patients undergoing robot-assisted radical cystectomy (AUC, 0.6041) [14]. Kayra et al. also reported favorable discriminative ability of E-PASS in patients undergoing open radical cystectomy [15]. E-PASS has additionally demonstrated favorable performance in other major surgical populations, including patients undergoing laparoscopic radical gastrectomy [16]. However, the present findings should not be interpreted as a conventional direct assessment of superiority. Although E-PASS CRS exhibited the highest documented AUC, its capacity for discrimination was not significantly different from that of SORT or the other evaluated scores. As a result, the present study does not establish that E-PASS exceeds other risk evaluation tools. The numerically higher AUC observed for E-PASS CRS in our cohort may partly reflect its broader incorporation of perioperative risk, because the CRS combines preoperative physiological reserve with intraoperative measures of surgical stress, including blood loss and operative time. Consequently, its performance should not be interpreted as evidence of superiority as a purely preoperative prediction tool. Rather, E-PASS may provide a more comprehensive estimate of perioperative risk by incorporating both patient-related vulnerability and the magnitude of surgical stress. In our cohort, operative time itself was not independently associated with major complications, suggesting that the performance of E-PASS was unlikely to be explained by operative duration alone.

SORT demonstrated an AUC close to that of E-PASS CRS and was associated with major complications in the univariable analysis. However, SORT was originally developed primarily to estimate postoperative mortality rather than postoperative morbidity, which may limit its discriminatory performance when major complications are used as the outcome. ASA classification, although simple and widely used, provides a broad assessment of systemic disease burden and does not directly incorporate procedure-specific characteristics or frailty. In our cohort, ASA class III was more frequent among patients who developed major complications, but ASA was not a predictor in the logistic regression models. The mFI-5 was associated with major complications in univariable analysis, although its discriminatory performance was also limited. The interpretation of this finding requires particular caution because functional dependence, one of the components of the mFI-5, was operationalized using ECOG performance status ≥2 rather than a standardized frailty or functional-dependence instrument. Formal geriatric and ADL/IADL assessments are often unavailable in retrospective surgical datasets; accordingly, ECOG performance status, which is routinely documented in oncologic care, has been used in previous surgical studies as a pragmatic surrogate for functional dependence [11,17,18,19,20]. Exploratory secondary analyses also suggested that the evaluated scores may capture different aspects of perioperative risk. E-PASS CRS was associated with 30-day readmission, whereas SORT was associated with 90-day mortality; ASA, E-PASS CRS, and mFI-5 showed only weak correlations with ICU length of stay. Because these analyses were based on relatively few events and were not adjusted for multiple comparisons, they should be regarded as exploratory and hypothesis-generating. These associations were not consistent across outcomes and should therefore be interpreted cautiously.

The loss of statistical significance when all four scores were entered simultaneously into the multivariable model likely reflects overlap in the clinical information captured by these tools. Several components are shared directly or indirectly across the scores, including age, ASA classification, comorbidity burden, functional status, and physiological reserve. Simultaneous inclusion of these related measures may therefore attenuate the apparent independent contribution of each score. Accordingly, the fully adjusted models should be interpreted as demonstrating overlapping risk information rather than as evidence that the individual scoring systems lack clinical relevance. In particular, the multivariable analysis was not intended to identify a single independently superior score, but rather to examine whether each score retained an association with major complications after accounting for the information represented by the other tools.

The modest discriminatory performance observed in our study also suggests that postoperative morbidity after radical cystectomy is unlikely to be adequately captured by a limited set of clinical variables. Several potentially relevant factors are not fully represented in the evaluated scores. Nutritional status, particularly hypoalbuminemia, has been associated with increased morbidity and mortality after radical cystectomy [21], while liver disease and impaired physiological reserve may further influence postoperative recovery [22]. Procedure-related factors, including the extent of lymph-node dissection, perioperative blood loss, and transfusion requirements, have also been associated with postoperative outcomes [23,24]. In addition, surgical approach, enhanced recovery protocols, and institutional volume may influence perioperative morbidity [25,26,27]. Incorporating such clinical, laboratory, functional, and procedure-specific variables may therefore improve individualized risk prediction. The relatively low AUC values observed across all four tools support the need for risk models specifically developed and validated for radical cystectomy rather than reliance on generic surgical risk scores alone.

More comprehensive prediction approaches may offer an alternative to conventional scoring systems. Recent machine-learning models integrating demographic, laboratory, and perioperative variables have shown promising discriminatory performance for short-term morbidity and mortality after radical cystectomy [28]. However, improved statistical performance does not necessarily translate into clinical association. Future models should therefore undergo external validation, calibration assessment, and evaluation of clinical applicability before routine implementation.

This study has several limitations. First, the retrospective and single-center design with the use of complete-case analysis may introduce selection bias due to the exclusion of patients with missing data, posing a risk of information bias and limiting the generalizability of the findings. Second, the evaluated risk scores were calculated retrospectively from routinely collected clinical data. Third, E-PASS CRS incorporates intraoperative variables and therefore differs conceptually from the predominantly preoperative ASA, SORT, and mFI-5 systems. This difference in prediction horizon should be considered when interpreting the comparative performance of the scores and precludes interpreting the findings as a direct comparison of exclusively preoperative prediction tools. Fourth, the retrospective nature of the study did not allow for the evaluation of functional dependence using standardized geriatric tools (such as the Katz ADL or Lawton IADL) and was defined by an ECOG performance status ≥ 2. In addition, potentially relevant variables, including nutritional parameters and the consistent application of enhanced recovery protocols, were not available for all patients. All patients underwent open radical cystectomy with ileal conduit urinary diversion, which reduced procedural heterogeneity but may limit the generalizability of our findings to robotic approaches or other diversion types. Finally, our secondary outcome analyses were underpowered due to the small sample size and low event rates for outcomes such as mortality. Although this provided a defined number of events for the primary analysis, the study was not sufficiently powered to draw definitive conclusions from secondary mortality and other low-frequency outcomes. Accordingly, secondary endpoint analyses should be considered exploratory and hypothesis-generating. Lastly, the limited sample size made visual calibration plots unattainable. Our results thus indicate discriminative ability rather than conclusive predictive performance, which is required to be validated in larger cohorts.

## 5. Conclusions

Among the four evaluated risk assessment tools, none demonstrated strong discriminatory performance for major postoperative complications after radical cystectomy. E-PASS CRS had the highest numerical AUC, but this difference was not statistically significant compared with SORT or the other evaluated scores. These findings suggest that existing risk models may support perioperative risk stratification but should not be used as stand-alone predictors of major postoperative morbidity. The findings also highlight the limitations of interpreting E-PASS CRS as directly equivalent to purely preoperative risk assessment tools, given its incorporation of intraoperative surgical-stress variables. More comprehensive models incorporating patient-related, functional, and procedure-specific factors may improve risk prediction in patients undergoing radical cystectomy. Future radical-cystectomy-specific models should incorporate patient-related, functional, nutritional, laboratory, and procedure-specific factors and undergo prospective external validation.

## Figures and Tables

**Table 1:** Baseline demographic, clinical, and perioperative characteristics according to the presence of major postoperative complications.

Variable	Overall Cohort (*n* = 193)	No Major Complications (*n* = 135)	Major Complications (*n* = 58)	*p*
Age, years	66 (59–72)	64 (58–72)	67 (61.3–72.8)	0.155
Male sex, *n* (%)	168 (87.0)	119 (88.1)	49 (84.5)	0.487
BMI, kg/m^2^	25.2 (22.9–28.0)	25.1 (22.7–27.7)	25.4 (23.7–28.7)	0.322
ECOG performance status ≥ 2, *n* (%)	21 (10.9)	14 (10.4)	7 (12.1)	0.728
ASA class III–IV, *n* (%)	112 (58.0)	69 (51.1)	43 (74.1)	0.003
Operative time, min	280 (230–325)	290 (230–325)	270 (240–325)	0.791
E-PASS Comprehensive Risk Score (CRS)	0.38 (0.21–0.56)	0.352 (0.203–0.531)	0.435 (0.278–0.628)	0.019
SORT predicted 30-day mortality risk, %	4.95 (1.50–10.14)	4.93 (1.25–10.14)	5.09 (2.68–10.14)	0.029
mFI-5 ≥ 2, *n* (%)	62 (32.1)	37 (27.4)	25 (43.1)	0.032
Clavien–Dindo classification, *n* (%) Grade I Grade II Grade III Grade IV Grade V	45 (23.3) 90 (46.6) 36 (18.7) 18 (9.3) 4 (2.1)	45 (33.3) 90 (66.7) 0 (0.0) 0 (0.0) 0 (0.0)	0 (0.0) 0 (0.0) 36 (62.1) 18 (31.0) 4 (6.9)	

Continuous variables are presented as median (interquartile range) and categorical variables as *n* (%). Continuous variables were compared using the Mann–Whitney U test; categorical variables were compared using the χ^2^ test or Fisher’s exact test, as appropriate.

**Table 2:** Logistic regression results for major postoperative complications (total sample: 193 patients).

Risk Indicator/Variable	1	2	3	4	5	6	7	8	9	10
E-PASS CRS OR (95% CI), *p*	4.48 (1.36–14.75), *p* = 0.013	4.44 (1.34–14.68), *p* = 0.014	-	-	-	-	-	-	1.73 (0.42–8.12), *p* = 0.336	1.55 (0.35–6.81), *p* = 0.462
SORT OR (95% CI), *p*	-	-	1.07 (1.00–1.15), *p* = 0.042	1.08 (1.00–1.16), *p* = 0.038	-	-	-	-	1.01 (0.92–1.11), *p* = 0.87	1.00 (0.91–1.10), *p* = 0.754
mFI-5 ≥ 2 (Ref: mFI-5 < 2) OR (95% CI), *p*	-	-	-	-	2.01 (1.05–3.84), *p* = 0.034	2.10 (1.09–4.04), *p* = 0.027	-	-	1.55 (0.71–3.41), *p* = 0.374	1.56 (0.71–3.45), *p* = 0.27
ASA II (Ref: ASA I) OR (95% CI), *p*	-	-	-	-	-	-	1.71 (0.19–15.65), *p* = 0.628	1.80 (0.19–16.92), *p* = 0.601	1.60 (0.17–15.02), *p* = 0.686	1.60 (0.17–15.07), *p* = 0.685
ASA III (Ref: ASA I) OR (95% CI), *p*	-	-	-	-	-	-	3.55 (0.41–31.02), *p* = 0.249	3.89 (0.44–34.19), *p* = 0.226	2.51 (0.27–23.36), *p* = 0.416	2.51 (0.27–23.47), *p* = 0.387
Operative time (Per 10 min) OR (95% CI), *p*	-	1.01 (1.00–1.02), *p* = 0.150	-	1.01 (1.00–1.02), *p* = 0.142	-	1.01 (1.00–1.02), *p* = 0.158	-	1.01 (1.00–1.02), *p* = 0.149	-	1.02 (1.00–1.04), *p* = 0.085

Data are presented as Odds Ratios (OR) with 95% Confidence Intervals (CI) and exact *p*-values. Columns (1), (3), (5), and (7) report univariable logistic regression models for each risk indicator. Columns (2), (4), (6), and (8) present the corresponding models additionally adjusted for operative time. Columns (9) and (10) present fully adjusted models incorporating all risk indicators simultaneously, without and with operative time, respectively, to assess potential collinearity and the robustness of the estimates. Reference categories were mFI-5 < 2 for mFI-5 ≥ 2; ASA physical status class I for ASA II and ASA III. Operative time is expressed in 10 min increments. E-PASS CRS and SORT scores are evaluated as continuous variables. OR, Odds Ratio; CI, Confidence Interval; Ref, Reference category; ASA, American Society of Anesthesiologists; mFI-5, 5-item Modified Frailty Index.

**Table 3:** ROC analysis of risk scores for major postoperative complications.

Risk Score	AUC	Standard Error	*p* Value	95% CI
E-PASS CRS	0.618	0.042	0.009	0.536–0.701
SORT	0.610	0.043	0.016	0.525–0.694
ASA	0.595	0.044	0.037	0.509–0.680
mFI-5 ≥ 2	0.578	0.046	0.084	0.489–0.668

Abbreviations: AUC, area under the receiver operating characteristic curve; CI, confidence interval; CRS, Comprehensive Risk Score; E-PASS, Estimation of Physiologic Ability and Surgical Stress; SORT, Surgical Outcome Risk Tool; mFI-5, modified 5-item Frailty Index.

**Table 4:** Associations of risk scores with secondary postoperative outcomes.

Postoperative Outcome	ASA ≥ 3	E-PASS CRS	SORT Risk	mFI-5 ≥ 2
ICU admission, OR (95% CI); *p*	1.46 (0.64–3.33); 0.374	1.00 (0.86–1.17); 0.997	1.05 (0.94–1.17); 0.360	1.51 (0.57–3.97); 0.407
ICU length of stay, ρ; *p*	0.147; 0.042	0.154; 0.033	0.132; 0.068	0.155; 0.032
Total hospital stay, ρ; *p*	−0.100; 0.166	−0.049; 0.494	−0.081; 0.265	−0.151; 0.036
30-day readmission, OR (95% CI); *p*	1.14 (0.53–2.44); 0.742	1.17 (1.01–1.34); 0.031	1.01 (0.92–1.10); 0.860	1.20 (0.54–2.67); 0.652
30-day mortality, OR (95% CI); *p*	NE	1.26 (0.97–1.64); 0.087	1.03 (0.87–1.22); 0.746	1.81 (0.39–8.37); 0.447
90-day mortality, OR (95% CI); *p*	1.98 (0.60–6.57); 0.262	1.03 (0.84–1.27); 0.756	1.15 (1.02–1.28); 0.017	0.37 (0.08–1.71); 0.204
1-year mortality, OR (95% CI); *p*	1.61 (0.77–3.40); 0.207	1.14 (1.00–1.30); 0.050	1.03 (0.95–1.12); 0.450	1.49 (0.70–3.14); 0.300

Binary outcomes were analyzed using univariable logistic regression and are presented as ORs with 95% CIs. ICU and total hospital length of stay were analyzed using Spearman correlation and are presented as ρ values. E-PASS CRS is expressed per 0.1-unit increase, SORT per 1-percentage-point increase in predicted 30-day mortality risk, and mFI-5 as ≥2 vs. <2. NE, not estimable. OR, odds ratio; CI, confidence interval; ICU, intensive care unit.

**Table 5:** Pairwise comparisons of ROC curves (DeLong test).

Scores	E-PASS CRS	SORT	ASA	mFI-5
E-PASS CRS	-	*p* = 0.841	*p* = 0.610	*p* = 0.356
SORT	*p* = 0.841	-	*p* = 0.638	*p* = 0.527
ASA	*p* = 0.610	*p* = 0.638	-	*p* = 0.739
mFI-5	*p* = 0.356	*p* = 0.527	*p* = 0.739	-

## Data Availability

The data presented in this study are available on request from the corresponding author.
